# Evaluation of analytical similarity between trastuzumab biosimilar CT-P6 and reference product using statistical analyses

**DOI:** 10.1080/19420862.2018.1440170

**Published:** 2018-03-14

**Authors:** Jihun Lee, Hyun Ah Kang, Jin Soo Bae, Kyu Dae Kim, Kyoung Hoon Lee, Ki Jung Lim, Min Joo Choo, Shin Jae Chang

**Affiliations:** Biotechnology Research Institute, R&D Division, Celltrion Inc., Incheon, Korea

**Keywords:** biosimilar, CT-P6, Herzuma^TM^, reference product, statistical analyses, trastuzumab

## Abstract

The evaluation of analytical similarity has been a challenging issue for the biosimilar industry because the number of lots for reference and biosimilar products available at the time of development are limited, whilst measurable quality attributes of target molecule are numerous, which can lead to potential bias or false negative/positive conclusions regarding biosimilarity. Therefore, appropriate statistical analyses are highly desirable to achieve a high level of confidence in the similarity evaluation. A recent guideline for the risk-based statistical approaches recommended by the US Food and Drug Administration provides useful tools to systematically evaluate analytical similarity of biosimilar products compared with reference products. Here, we evaluated analytical similarity of CT-P6, a biosimilar product of trastuzumab, with the reference products (EU-Herceptin® or US-Herceptin®) following these statistical approaches. Various quality attributes of trastuzumab were first ranked based on the clinical impact of each attribute and subsequently adjusted to one of three tiers (Tier 1, Tier 2 and Tier 3) considering the characteristics of the assay, the level of attribute present and the feasibility of statistical analysis. Two biological activities with highest potential clinical impact were evaluated by an equivalent test (Tier 1), and other bioactivities and structural/physicochemical properties relevant to the clinical impact were evaluated by a quality range approach (Tier 2). The attributes with low risk ranking or qualitative assay were evaluated by visual comparison (Tier 3). Analytical similarity assessment analyzed by the three tiers clearly demonstrated that CT-P6 exhibits highly similar structural and physicochemical properties, as well as functional activities, compared with the reference products. There were small differences observed in a few quality attributes between CT-P6 and the reference products, but the differences were very minor, and unlikely to impact on clinical outcome. The recently reported equivalent clinical efficacy of CT-P6 with the reference product further supports that CT-P6 is highly similar compared with the reference product in the view of totality-of-evidence.

## Introduction

A biosimilar, also referred to as a follow-on-biologic, is a product that shows no clinically meaningful difference from approved biologics. Compared to developing generic of chemical drug, developing biosimilar product is challenging due to large size and inherent complexity of biologics which is produced by living cells. As the quality of biologics is process-dependent and most critical information for manufacturing and analysis remains confidential, developers must use advanced technology from initial development to the final quality assessment of the product to be marketed. Abbreviated licensure pathway for biosimilar is provided based on the notion that biosimilarity has been demonstrated.^1–4^ Although details vary among countries, guidelines commonly indicate that no difference in potential clinical impact in terms of safety, purity and potency should be shown between a biosimilar product and a reference product, using orthogonal and state-of-the-art assays that can fully identify the physico-chemical and biological characteristics of the product. Accordingly, several biosimilar products have shown their analytical biosimilarity with reference products.^5–10^

CT-P6 was developed as a biosimilar of Herceptin® (trastuzumab) by Celltrion Inc., and recently received a positive opinion for market authorization by the European Medicines Agency (EMA)'s Committee for Medicinal Products for Human Use.^11^ Herceptin® is approved for the treatment of patients with certain breast and metastatic gastric cancers that overexpress human epidermal growth factor receptor 2 (HER2) by the US Food and Drug Administration (FDA) and EMA.^12^^,^^13^ Herceptin® has been shown to inhibit the uncontrolled growth of cancer cells via binding to the extracellular domain of HER2 in breast cancers that express elevated levels of HER2.^14^ Also, like other monoclonal antibodies (mAbs) used for cancer treatment, it can kill cancer cells through antibody-dependent cell-mediated cytotoxicity (ADCC).^15–18^ Two other trastuzumab biosimilar products (Ontruzant®, developed by Samsung Bioepis and Ogivri™, developed by Mylan and Biocon) have received approval recently in the European Union (EU) and United States (US), respectively.^19^^,^^20^

Because the biosimilarity is assessed in terms of totality-of-evidence, statistical evaluation for the assay is critical. It is well known that the original products exhibit lot-to-lot heterogeneity in certain quality attributes.^21–24^ Therefore, assessment should be performed with an adequate number of meaningful lots and attributes/assays should be considered. For determining a statistical evaluation method, FDA recommended a risk-based approach.^25^ They suggested that the analytical similarity assessment should be planned with risk ranking of the reference product's quality attributes based on the potential impact on the clinical outcome, and then the statistical method should be applied among three tiers with appropriate similarity acceptance criteria. Equivalence testing (Tier 1) is applied for attributes with the highest potential clinical impact and assays that explain clinically relevant mechanism of action of the drug. Two one-sided tests on the mean difference of developing and the reference products are used, and use of an interval (−1.5$\times \sigma_{R}$, 1.5$\times \sigma_{R}$) that can support 90% confidence interval is recommended. A quality ranges approach (Tier 2) is recommended for attributes with lower risk rank. Standard deviation of the reference product is used to determine similarity acceptance criteria, and the multiplier for standard deviation should be justified based on the scientific background. Evaluation through visual comparison (Tier 3) is recommended for attributes with the lowest risk ranking and test of qualitative results. According to FDA's guideline, in addition to risk ranking, other factors such as level of attributes, tier of other orthogonal assays for the same attributes, and character of assays should be considered in determining tier.^25^

Herein, we evaluated the analytical similarity of CT-P6 to the reference product Herceptin® in two regions (US and EU) following the tier approach. The results demonstrate that CT-P6 has highly similar structural/physicochemical properties and biological activities compared with EU-Herceptin® and US-Herceptin®. Our experience shows that statistical evaluation using the tier approach is a useful tool to systematically evaluate analytical similarity results.

## Results

As outlined in the various regulatory guidelines on the development of biosimilar products,^1–4^^,^^26^ a step-wise approach has been taken with respect to the demonstration of similarity of CT-P6 to Herceptin®, starting with a comprehensive physicochemical and biological characterization of CT-P6 relative to its reference product. A wide range of state-of-the-art orthogonal methodologies were used to compare physicochemical properties and biological activities of CT-P6, EU- and US-Herceptin®. The investigated attributes include the primary structure, higher order structure, purity/impurity profiles, charged variants, glycan structures, as well as various aspects of product functionalities. The evaluation of the analytical similarity was conducted as described below.

### Risk ranking

In order to score risk ranking based on the clinical impact of each attribute, the guideline suggests consideration of at least two factors: 1) potential impact of an attribute on clinical performance, and 2) the degree of uncertainty around a certain quality attribute.^25^ Accordingly, we developed a risk assessment strategy with five levels (very high → high → medium → low → very low) of “potential clinical impact” and three levels (high → medium → low) of “degree of uncertainty” (Table 1). Prior knowledge from literature reports and the results of *in-house* studies were utilized to determine the levels for these two factors. For example, the level of “potential clinical impact” selected was “very high” if an attribute directly affects clinical outcome (e.g., potency, pharmacokinetics (PK)/pharmacodynamics (PD), safety, and immunogenicity). The level of “degree of uncertainty” was determined to be “high” if there is limited understanding of the clinical impact of an attribute. Risk rankings for each quality attribute were subsequently determined by multiplying the scores of “potential clinical impact” and “degree of uncertainty”. In general, biological activities were given greater weight on the risk ranking than physicochemical properties since they directly measure activities linked to mechanisms of action, activity, efficacy, safety and immunogenicity of the product.

**Table 1.** Risk ranking determination and tier classification.

Potential Clinical Impact		Degree of Uncertainty		Risk Ranking	Tier
Very High,		High,		Very High	1
High,		Medium,		High	2
Medium,	×	Low	=	Moderate	
Low,				Low	3
Very Low				Very Low	

For example, risk ranking for anti-proliferation activity, HER2 binding affinity and ADCC activity were determined to be “very high” since they are key mechanisms of action of trastuzumab for its clinical efficacy as a treatment for breast cancer. Other clinically relevant biological activities were ranked either “high” or “moderate” depending on their significance on clinical efficacy. Other biological activities such as C1q binding affinity were ranked “low” since they have little role in the antitumor activity of trastuzumab.

With regards to the physicochemical properties, primary amino acid sequence that is directly related to efficacy and safety of the product were ranked “high”. Purity related attributes such as aggregates and fragments were ranked “moderate” considering they may cause reduced biological activity and immunogenicity. Selected quality attributes, including heavy chain (HC) Asp102 isomerization, non-glycosylation, afucosylation and high mannose, that have been known to affect biological activity of the product were also ranked “moderate”. Risk ranking of the rest of the quality attributes such as C-terminal lysine variants were ranked “low” or “very low” since they have little impact on clinical efficacy or safety.

### Tier classification

Based on the risk ranking, the level of tier was subsequently adjusted considering the characteristics of the assay, including its sensitivity, the level of attribute present and the feasibility of statistical analysis.^25^ Tier 1 statistical analysis was carried out using the equivalence test for quality attributes with “very high” risk ranking that directly evaluate clinically relevant mechanisms of action (e.g., anti-proliferation or ADCC) of the product. Tier 2 statistical analysis was carried out using the quality range approach to assess the quality attributes with the “high” to “moderate” risk ranking, for which quantitative data can be obtained. Tier 3, an evaluation by raw or graphical data comparison, was applied for the quality attributes with “low” to “very low” risk ranking. Tier 3 was also assigned for attributes where statistical analysis is not feasible. The strategy of tier classification is shown in Table 1, and the assigned tier for each quality attribute is listed in Table 2.

**Table 2.** Quality Attributes and Test Methods used for Physicochemical and Biological Similarity Assessment between CT-P6 and Herceptin®.

Attribute		Risk Ranking	Test/Assay	Measurement	Tier of Statistical Analysis
Primary Structure	Primary Amino Acid Sequence	High	Molar Absorptivity	Molar absorptivity/ Extinction coefficient	2
			Peptide Mapping (HPLC)	Peak profile	3^1^
			Peptide Mapping (LC-MS)	Peak profile	3^1^
			N-terminal Sequencing	Sequence identity	3^1^
			C-terminal Sequencing	Sequence identity	3^1^
Post Translational	Deamidation	Moderate	Peptide Mapping (LC-MS)	% Deamidation (Asn)	2
Modifications	Oxidation	Low	Peptide Mapping (LC-MS)	% Oxidation (Met)	3
	Isomerization	Moderate	Peptide Mapping (LC-MS)	% Isomerization (Asp)	2
	N-terminal Glutamine	Very Low	Peptide Mapping (LC-MS)	% N-terminal glutamine	3
	C-terminal Lysine Truncation	Very Low	Peptide Mapping (LC-MS)	% C-terminal lysine	3
	C-terminal Proline Amidation	Very Low	Peptide Mapping (LC-MS)	% C-terminal proline amidation	3
Higher Order Structure	Disulfide Bonds	Moderate	Native and Reduced Peptide Mapping	Disulfide bond positions	3
			Free Thiol Analysis	Free thiol (SH groups)	2
	Secondary Structure		FTIR	Structure of protein by spectroscopy	3^1^
	Secondary and Tertiary Structure		CD	a-helical, b-sheet and unordered structures	3^1^
	Thermal Stability		DSC	Thermal unfolding temperatures	2
	Tertiary Structure		Antibody Array	3D conformational epitope exposure	3^1^
Purity/Impurity	Aggregates	Moderate	SEC-HPLC	% Monomer content	2
				% HMW Content	2
			SEC-MALS	Monomer size (kDa)	2
				HMW size (kDa)	3^2^
				% Monomer content	2
				% HMW content	2
			AUC	Monomer S value	2
				Dimer S value	2
				% Monomer content	2
				% Dimer content	2
	Fragments	Moderate	Non-reduced CE-SDS	% Intact IgG	2
				% Sum of non-Assembled Fragments	2
	HCP	Moderate	Residual Host Cell Protein ELISA	HCP (ppm)	3
	Host Cell DNA	Moderate	Residual Host Cell DNA PCR	DNA (ppb)	3
	rProteinA	Moderate	Residual rProtein A ELISA	rProtein A (ppm)	3
	Sub-visible	Moderate	MFI	1≤, <100 (μm)	3
	particles			2≤, <100 (μm)	
				5≤, <100 (μm)	
				10≤, <100 (μm)	
				15≤, <100 (μm)	
				25≤, <100 (μm)	
				40≤, <100 (μm)	
				50≤, <100 (μm)	
				70≤, <100 (μm)	
				(Cumulative Counts/mL)	
Charge Variants	Charge Variants	Moderate (Deamidated/Isomerization)	IEF	pI values of charge variants	3^1^
		Very Low (All others)	IEC-HPLC	% Peak 1+Peak 2+Peak 3+Peak 4	2
				% Peak 6	2
				% Peak 5	3
				% Peak 7	
Glycosylation	Non-glycosylated Product	Moderate	Reduced CE-SDS	% Non-glycosylated Heavy Chain % L+H	2
	Afucosylated Glycans	Moderate	Oligosaccharide Profiling	%G0+G1+G2	2
			N-linked Glycan Analysis	%G0+G1+G2	2
	High Mannose Glycans	Moderate	Oligosaccharide Profiling	%Man5+Man6+ Man8	2
			N-linked Glycan Analysis	%Man5	2
	Total Afucosylated Glycans	Moderate	Oligosaccharide Profiling	%G0+G1+G2+Man5+Man6+Man8	2
			N-linked Glycan Analysis	%G0+G1+G2+ Man5	2
	Galactosylated	Low	Oligosaccharide Profiling	Agalactosylated:	3
	Glycans			% G0F,	
				%G0F-GN	
				Galactosylated:	3
				%[G1F-GN]+G1F+G2F	
			N-linked Glycan Analysis	Agalactosylated:	3
				% G0F	
				Galactosylation:	3
				%G1F+G2F	
	Sialic Acids	Low	Oligosaccharide Profiling	%[G1F-GN+NANA]+[G1F+NANA]+[G2F+NANA]+[G2F+2NANA]	3
			Sialic Acid Analysis	NANA (sialic acid / protein, mol / mol)	3
	Glycation	Low	Glycation analysis	% Glycation at light chain	3
				% Glycation at heavy chain	
Fab Binding	HER2 Binding	Very High	HER2 Binding Affinity (ELISA)	Relative HER2 Binding (%)	2^3^
			Cell-based HER2 Binding Affinity (CELISA)		2^3^
	Anti-proliferation	Very high	In Vitro Bioactivity (anti-proliferation) using BT-474 Cell	Relative Anti-proliferation (%)	1
Fc Binding	C1q Binding	Low	C1q Binding (ELISA)	Relative C1q Binding (%)	3
	FcγRIIIa Binding	High	FcγRIIIa V Type Binding Affinity (SPR)	Relative FcγRIIIa V Type Binding Affinity (%)	2
			FcγRIIIa F Type Binding Affinity (SPR)	Relative FcγRIIIa F Type Binding Affinity (%)	2
	FcγRIIIb Binding	Moderate	FcγRIIIb Binding Affinity (SPR)	Relative FcγRIIIb Binding Affinity (%)	2
	FcγRIIa Binding	High	FcγRIIa Binding Affinity (SPR)	Relative FcγRIIa Binding Affinity (%)	2
	FcγRIIb Binding	Moderate	FcγRIIb Binding Affinity (SPR)	Relative FcγRIIb Binding Affinity (%)	2
	FcγRI Binding	Low	FcγRI Binding Affinity (SPR)	Relative FcγRI Binding Affinity (%)	3
	FcRn Binding	Moderate	FcRn Binding Affinity (SPR)	Relative FcRn Binding Affinity (%)	2
Fab –Fc Mediated Activities	ADCC	Very High	ADCC (PBMC)	Relative ADCC Potency (%)	1

1 Tier 3 was assigned because nature of the assays is qualitative despite of “high” or “moderate” risk ranking.

2 Tier 3 was assigned due to the trace amount of HMW content to precisely evaluate the molecular weight by MALS.

3 Tier 2 was assigned considering HER2 binding affinity does not measure the MoAs (anti-proliferation or ADCC activities) directly relevant to the clinical efficacy of trastuzumab.

### Similarity evaluation by Tier 1

Tier 1 equivalence testing is applied for attributes with the highest potential clinical impact and assays that explain clinically relevant mechanism of action of the drug. In the quality attributes of trastuzumab, anti-proliferation activity and ADCC activity were selected as Tier 1 (Table 2).

Trastuzumab binds to the extracellular domain of HER2, and has been shown to selectively exert anti-tumor effects in cancer models and patients with HER2-amplified breast cancer. Trastuzumab binding to HER2 blocks proteolytic cleavage of the extracellular domain of HER2, resulting in diminished levels of the more active p95–HER2 form of HER2 and selectively inhibiting ligand-independent HER2 interactions, including HER2-HER3 heterodimerization, leading to cell cycle arrest and reducing proliferation.^27–30^ As a result of these effects on the HER2 receptor, trastuzumab causes downregulation of PI3K-Akt-mTOR pathway signaling and regulators of cell cycle progression such as cyclin-dependent kinase inhibitor p27kip1 and cyclin D1.^30^^,^^31^ As PI3K-Akt-mTOR signaling relate to proliferation, and tumor growth and invasion is a major effector of HER2 activity, the blockade of the PI3K signaling pathway by trastuzumab suppresses tumor growth in multiple models of HER2-overexpressing breast cancer.^32^^,^^33^ Therefore, anti-proliferation activity plays a critical role in a mechanism of action of trastuzumab in HER2-overexpressing cancer.

ADCC activity of trastuzumab is mediated by the interaction of the Fc region with Fcγ receptors, which are expressed on various immune effector cells (macrophages/monocytes and natural killer (NK) cells), but especially FcγRIIIa (CD16).^34^ ADCC associated with trastuzumab treatment has been demonstrated in numerous breast cancer cell lines.^15^^,^^16^ Thus, ADCC is considered to be a key mechanism of action of trastuzumab in anti-tumor effects, and NK cells within peripheral blood mononuclear cells (PBMCs) play a key role in the ADCC effect of trastuzumab.^17^^,^^18–35^

**Figure 1.**
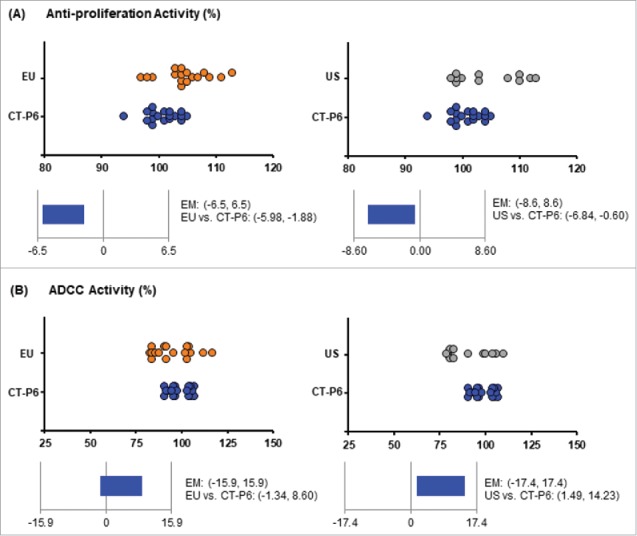
Scatter plots and equivalence test results for Tier 1 quality attributes. Comparisons of “CT-P6 *vs*. EU-Herceptin®” and “CT-P6 *vs*. US-Herceptin®” are shown in left and right, respectively. (A) Scatter plots (up) of relative anti-proliferation activity for CT-P6 (blue dot), EU-Herceptin® (orange dot) and US-Herceptin® (grey dot). Equivalence test results (bottom) are shown with 90% CI of mean difference between the 2 products. (B) Scatter plots (up) of relative ADCC activity for CT-P6 (blue dot), EU-Herceptin® (orange dot) and US-Herceptin® (grey dot). Equivalence test results (bottom) are shown with 90% CI of mean difference between the 2 products.

The results of the relative anti-proliferation activity and relative ADCC activity of CT-P6, EU- and US-Herceptin® are shown in Table 3. Fig. 1A present scatter plots of the anti-proliferation activity and their equivalence test results comparing CT-P6 vs. EU-Herceptin® as well as CT-P6 vs. US-Herceptin®. The results demonstrate that the 90% confident interval (CI) of mean difference between 2 products are within the equivalence margin (EM), indicating that CT-P6 and Herceptin® are statistically equivalent and thus have highly similar potency in terms of anti-proliferation activity. Scatter plots of the ADCC activity for CT-P6 and Herceptin® are shown in Fig. 1B. The equivalence test results revealed the 90% CI of mean difference between 2 products are within the EM, suggesting that CT-P6 has statistically equivalent ADCC activity with Herceptin®.

**Table 3.** Summary of Similarity Assessment Results for Biological Assays.

					Similarity Evaluation (EM^1^ or QR^2^)	
Quality Attribute / Test Method		Product	Min – Max	Mean ± SD	EU vs. CT-P6	US vs. CT-P6
Tier 1						
F(ab’) related Activities^3^	Anti-proliferation	EU-Herceptin®	97 – 113	105 ± 4.3	Within EM	Within EM
		CT-P6	94 – 105	101 ± 2.8		
		US-Herceptin®	98 – 113	105 ± 5.7		
Fab-Fc Mediated Activities^3^	ADCC (%)	EU-Herceptin®	83 – 117	96 ± 10.6	Within EM	Within EM
		CT-P6	91 – 107	99 ± 5.8		
		US-Herceptin®	79 – 110	91 ± 11.6		
Tier 2						
F(ab’) Binding^3^	HER2 binding affinity (ELISA)	EU-Herceptin®	92 – 103	97 ± 3.3	100.0% QR	100.0% QR
		CT-P6	94 – 106	100 ± 4.0		
		US-Herceptin®	95 – 112	100 ± 4.9		
	Cell-based HER2 binding affinity (CELISA)	EU-Herceptin®	87 – 113	99 ± 7.3	100.0% QR	100.0% QR
		CT-P6	90 – 116	100 ± 8.8		
		US-Herceptin®	87 – 114	102 ± 9.3		
Fc Binding^3^	FcγRIIIa-V binding affinity (SPR)	EU-Herceptin®	73 – 103	90 ± 9.2	100.0% QR	100.0% QR
		CT-P6	90 – 102	96 ± 3.4		
		US-Herceptin®	71 – 110	87 ± 11.1		
	FcγRIIIa-F binding affinity (SPR)	EU-Herceptin®	70 – 105	91 ± 11.6	100.0% QR	100.0% QR
		CT-P6	96 – 104	99 ± 2.2		
		US-Herceptin®	80 – 101	90 ± 7.3		
	FcγRIIIb binding affinity (SPR)	EU-Herceptin®	67 – 108	89 ± 12.0	100.0% QR	100.0% QR
		CT-P6	87 – 105	97 ± 4.4		
		US-Herceptin®	66 – 106	83 ± 13.7		
	FcγRIIa binding affinity (SPR)	EU-Herceptin®	94 – 102	99 ± 2.1	100.0% QR	100.0% QR
		CT-P6	98 – 103	100 ± 1.4		
		US-Herceptin®	94 – 100	97 ± 2.2		
	FcγRIIb binding affinity (SPR)	EU-Herceptin®	90 – 104	96 ± 3.9	100.0% QR	100.0% QR
		CT-P6	95 – 102	99 ± 2.0		
		US-Herceptin®	91 – 103	95 ± 3.9		
	FcRn binding affinity (SPR)	EU-Herceptin®	93 – 105	99 ± 3.1	100.0% QR	100.0% QR
		CT-P6	95 – 102	100 ± 2.0		
		US-Herceptin®	93 – 105	99 ± 4.0		
Tier 3						
Fc Binding^3^	C1q binding affinity (ELISA)	EU-Herceptin®	89 – 113	100 ± 6.2	Highly similar	Highly similar
		CT-P6	92 – 115	104 ± 7.1		
		US-Herceptin®	93 – 110	102 ± 6.8		
	FcγRI binding affinity (SPR)	EU-Herceptin®	95 – 102	99 ± 2.3	Highly similar	Highly similar
		CT-P6	93 – 102	98 ± 2.4		
		US-Herceptin®	95 – 101	97 ± 1.9		

1 EM was determined as 1.5σR of Herceptin® data and results were determined as 90% CI of mean difference between two products.

2 The QR limits were set based on the range of the values obtained for the reference product variation, expressed as 3 times Standard Deviation (SD). High similarity was considered to have been demonstrated if 90% of data points were within the QR of Herceptin® lots.

3 Relative potency (%) or binding affinity (%) in comparison to CT-P6 in-house reference standard.

### Similarity evaluation by Tier 2

The Tier 2 quality ranges approach is used for the attributes with lower risk rank. Summaries of analytical results and similarity evaluation for Tier 2 attributes for biological and physicochemical assays are shown in Table 3 and Table 4, respectively.

**Table 4.** Summary of Similarity Assessment Results for Physicochemical Tests.

						Similarity Evaluation ^1^	
Quality Attribute / Test Method			Product	Min – Max	Mean ± SD	EU vs. CT-P6	US vs. CT-P6
Tier 2							
Primary structure	Molar Absorptivity	Molar Absorptivity (L∙mol^−1^∙cm^−1^)	EU-Herceptin®	203,104 – 227,138	213,172 ± 6,750.8	100.0% QR	100.0% QR
			CT-P6	202,824 – 222,010	212,741 ± 5,024.8		
			US-Herceptin®	202,999 – 220,972	212,833 ± 4,626.5		
		Extinction Coefficient (L∙g^−1^∙cm^−1^)	EU-Herceptin®	1.40 – 1.56	1.47 ± 0.047	100.0% QR	100.0% QR
			CT-P6	1.40 – 1.53	1.47 ± 0.035		
			US-Herceptin®	1.40 – 1.52	1.47 ± 0.031		
Post-translational modification	Peptide Mapping (LC-MS)	Deamidation at LC Asn30 (%)	EU-Herceptin®	6.7 – 10.5	8.8 ± 0.94	100.0% QR	55.6% QR
			CT-P6	8.6 – 9.5	9.0 ± 0.26		
			US-Herceptin®	9.2 – 10.2	9.8 ± 0.31		
		Isomerization at HC Asp102 (%)	EU-Herceptin®	2.4 – 7.7	4.0 ± 1.25	100.0% QR	100.0% QR
			CT-P6	2.7 – 6.5	4.2 ± 1.11		
			US-Herceptin®	2.7 – 7.9	4.8 ± 1.34		
Higher order structure	Free Thiol Analysis	Average (free SH/IgG, µM/µM)	EU-Herceptin®	0.25 – 0.36	0.31 ± 0.029	100.0% QR	72.2% QR
			CT-P6	0.25 – 0.35	0.28 ± 0.023		
			US-Herceptin®	0.29 – 0.34	0.31 ± 0.015		
	DSC (°C)	Tm1	EU-Herceptin®	71.0 – 71.4	71.2 ± 0.10	94.4% QR	100.0% QR
			CT-P6	70.9 – 71.3	71.1 ± 0.09		
			US-Herceptin®	71.0 – 71.5	71.1 ± 0.14		
		Tm2	EU-Herceptin®	81.0 – 81.4	81.1 ± 0.14	100.0% QR	94.4% QR
			CT-P6	81.0 – 81.4	81.1 ± 0.10		
			US-Herceptin®	81.0 – 81.2	81.1 ± 0.08		
		Tm3	EU-Herceptin®	82.9 – 83.2	83.0 ± 0.11	100.0% QR	94.4% QR
			CT-P6	82.9 – 83.2	83.0 ± 0.08		
			US-Herceptin®	82.9 – 83.1	82.9 ± 0.06		
Purity/Impurity	SEC-HPLC	Monomer (%)	EU-Herceptin®	99.5 – 99.7	99.6 ± 0.09	100.0% QR	100.0% QR
			CT-P6	99.6 – 99.7	99.7 ± 0.05		
			US-Herceptin®	99.5 – 99.8	99.6 ± 0.09		
		HMW (%)	EU-Herceptin®	0.3 – 0.5	0.4 ± 0.09	100.0% QR	100.0% QR
			CT-P6	0.2 – 0.3	0.3 ± 0.04		
			US-Herceptin®	0.2 – 0.5	0.4 ± 0.08		
	SEC-MALS	Monomer (%)	EU-Herceptin®	99.3 – 99.6	99.5 ± 0.09	100.0% QR	100.0% QR
			CT-P6	99.4 – 99.7	99.6 ± 0.08		
			US-Herceptin®	99.4 – 99.7	99.5 ± 0.10		
		HMW (%)	EU-Herceptin®	0.3 – 0.6	0.4 ± 0.08	100.0% QR	100.0% QR
			CT-P6	0.2 – 0.4	0.3 ± 0.07		
			US-Herceptin®	0.2 – 0.5	0.4 ± 0.09		
		Monomer (MW, kDa)	EU-Herceptin®	137 – 142	139 ± 1.2	100.0% QR	100.0% QR
			CT-P6	138 – 141	139 ± 0.9		
			US-Herceptin®	137 – 141	139 ± 1.4		
	AUC	Monomer (%)	EU-Herceptin®	97.4 – 98.2	97.8 ± 0.24	100.0% QR	88.9% QR
			CT-P6	97.2 – 98.3	97.8 ± 0.25		
			US-Herceptin®	97.5 – 97.9	97.7 ± 0.14		
		Dimer (%)	EU-Herceptin®	1.8 – 2.6	2.2 ± 0.24	100.0% QR	88.9% QR
			CT-P6	1.7 – 2.5	2.1 ± 0.21		
			US-Herceptin®	2.1 – 2.5	2.3 ± 0.14		
		Monomer (s-value)	EU-Herceptin®	5.1 – 5.3	5.2 ± 0.07	100.0% QR	100.0% QR
			CT-P6	5.1 – 5.3	5.2 ± 0.06		
			US-Herceptin®	5.1 – 5.2	5.2 ± 0.05		
		Dimer (s-value)	EU-Herceptin®	7.3 – 7.6	7.5 ± 0.09	100.0% QR	88.9% QR
			CT-P6	7.4 – 7.7	7.5 ± 0.09		
			US-Herceptin®	7.4 – 7.6	7.5 ± 0.06		
	CE-SDS (non-reduced)	Intact IgG (%)	EU-Herceptin®	98.0 – 98.8	98.3 ± 0.25	66.7% QR	66.7% QR
			CT-P6	96.8 – 98.3	97.7 ± 0.39		
			US-Herceptin®	97.7 – 98.5	98.2 ± 0.23		
		Sum of fragments (%)	EU-Herceptin®	1.2 – 2.1	1.7 ± 0.25	66.7% QR	72.2% QR
			CT-P6	1.7 – 3.3	2.3 ± 0.40		
			US-Herceptin®	1.5 – 2.3	1.9 ± 0.23		
Charge variants	IEC-HPLC	Acidic Group (Peak 1+2+3+4) (%)	EU-Herceptin®	20.2 – 31.4	26.5 ± 3.09	100.0% QR	100.0% QR
			CT-P6	26.3 – 31.1	28.3 ± 1.81		
			US-Herceptin®	27.0 – 31.3	29.1 ± 1.73		
		Peak 6 (%)	EU-Herceptin®	4.3 – 6.4	5.6 ± 0.85	100.0% QR	100.0% QR
			CT-P6	3.2 – 5.0	4.2 ± 0.65		
			US-Herceptin®	4.2– 6.3	5.2 ± 0.84		
Glycosylation	CE-SDS (reduced)	H+L (%)	EU-Herceptin®	99.1 – 99.6	99.4 ± 0.11	83.3% QR	88.9% QR
			CT-P6	98.8 – 99.5	99.3 ± 0.20		
			US-Herceptin®	99.1 – 99.6	99.3 ± 0.14		
		Non-glycosylated heavy chain (%)	EU-Herceptin®	0.5 – 0.9	0.6 ± 0.10	77.8% QR	88.9% QR
			CT-P6	0.5 – 1.2	0.8 ± 0.20		
			US-Herceptin®	0.4 – 0.9	0.7 ± 0.14		
	Oligosaccharide Profiling by 2-AB labeled HILIC-UPLC	Afucosylation (%)	EU-Herceptin®	3.1 – 7.9	5.1 ± 1.14	100.0% QR	100.0% QR
			CT-P6	4.6 – 6.6	5.3 ± 0.49		
			US-Herceptin®	3.2 – 6.7	4.7 ± 1.09		
		High Mannose (%)	EU-Herceptin®	1.3 – 3.1	2.2 ± 0.52	100.0% QR	100.0% QR
			CT-P6	2.1 – 3.4	2.6 ± 0.34		
			US-Herceptin®	1.7 – 4.1	2.3 ± 0.68		
		Total Afucosylation (%)	EU-Herceptin®	4.6 – 11.1	7.3 ± 1.59	100.0% QR	100.0% QR
			CT-P6	6.8 – 10.0	7.9 ± 0.77		
			US-Herceptin®	4.9 – 10.8	6.9 ± 1.57		
	N-linked Glycan Analysis by LC-MS	Afucosylation (%)	EU-Herceptin®	6.2 – 10.7	8.6 ± 1.34	100.0% QR	100.0% QR
			CT-P6	7.2 – 11.0	9.7 ± 0.86		
			US-Herceptin®	5.1 – 9.9	7.6 ± 1.87		
		High Mannose (%)	EU-Herceptin®	1.1 – 2.4	1.6 ± 0.38	100.0% QR	100.0% QR
			CT-P6	1.2 – 1.6	1.4 ± 0.12		
			US-Herceptin®	1.2 – 2.3	1.6 ± 0.33		
		Total Afucosylation (%)	EU-Herceptin®	7.5 – 13.2	10.2 ± 1.65	100.0% QR	100.0% QR
			CT-P6	8.8 – 12.6	11.2 ± 0.88		
			US-Herceptin®	6.3 – 11.6	9.2 ± 1.81		
Tier 3							
Primary structure	Peptide Mapping (HPLC)	Peak profile	EU-Herceptin®	Not Applicable	Not Applicable	Highly similar	Highly similar
			CT-P6				
			US-Herceptin®				
	Peptide Mapping (LC-MS)	Peak profile	EU-Herceptin®	Not Applicable	Not Applicable	Highly similar	Highly similar
			CT-P6				
			US-Herceptin®				
	N-terminal sequencing	Sequence identity	EU-Herceptin®	Not Applicable	Not Applicable	Highly similar	Highly similar
			CT-P6				
			US-Herceptin®				
	C-terminal sequencing	Sequence identity	EU-Herceptin®	Not Applicable	Not Applicable	Highly similar	Highly similar
			CT-P6				
			US-Herceptin®				
Post-translational modification	Peptide Mapping (LC-MS)	% Oxidation at HC Met255	EU-Herceptin®	1.5 – 2.5	1.8 ± 0.24	Highly similar	Highly similar
			CT-P6	1.8 – 2.5	2.1 ± 0.25		
			US-Herceptin®	1.7 – 2.3	2.0 ± 0.21		
		% N-terminal pyroglutamate at HC Glu01	EU-Herceptin®	1.2 – 2.1	1.7 ± 0.33	Highly similar	Highly similar
			CT-P6	1.4 – 1.7	1.5 ± 0.10		
			US-Herceptin®	1.8 – 2.2	1.9 ± 0.14		
		% C-terminal clipped lysine	EU-Herceptin®	97.9 – 99.0	98.6 ± 0.27	Highly similar	Highly similar
			CT-P6	95.8 – 96.8	96.2 ± 0.26		
			US-Herceptin®	98.5 – 99.1	98.9 ± 0.20		
		% C-terminal proline amidation	EU-Herceptin®	0.1 – 0.4	0.2 ± 0.12	C-terminal proline amidation in CT-P6 is higher than Herceptin®	C-terminal proline amidation in CT-P6 is higher than Herceptin®
			CT-P6	2.2 – 3.1	2.7 ± 0.23		
			US-Herceptin®	0.1 – 0.3	0.2 ± 0.04		
Higher order	Disulfide bond positioning		EU-Herceptin®	Not Applicable	Not Applicable	Highly similar	Highly similar
structure			CT-P6				
			US-Herceptin®				
	Secondary structure by FT-IR		EU-Herceptin®	Not Applicable	Not Applicable	Highly similar	Highly similar
			CT-P6				
			US-Herceptin®				
	Secondary and Tertiary structure by CD		EU-Herceptin®	Not Applicable	Not Applicable	Highly similar	Highly similar
			CT-P6				
			US-Herceptin®				
	3D epitope exposure by Antibody Array		EU-Herceptin®	Not Applicable	Not Applicable	Highly similar	Highly similar
			CT-P6				
			US-Herceptin®				
Purity/Impurity	SEC-MALS	HMW size (kDa)	EU-Herceptin®	Not Applicable	Not Applicable	Highly similar	Highly similar
			CT-P6				
			US-Herceptin®				
	Residual Host Cell Protein ELISA	HCP (ppm)	EU-Herceptin®	Not Applicable	Not Applicable	Highly similar	Highly similar
			CT-P6				
			US-Herceptin®				
	Residual Host Cell DNA PCR	HCP (ppb)	EU-Herceptin®	Not Applicable	Not Applicable	Highly similar	Highly similar
			CT-P6				
			US-Herceptin®				
	Residual rProtein A ELISA	rProtein A (ppm)	EU-Herceptin®	Not Applicable	Not Applicable	Highly similar	Highly similar
			CT-P6				
			US-Herceptin®				
	Sub-visible particles by MFI		EU-Herceptin®	Not Applicable	Not Applicable	Highly similar	Highly similar
			CT-P6				
			US-Herceptin®				
Charge variants	IEF	pI values of charge variants	EU-Herceptin®	Not Applicable	Not Applicable	Highly similar	Highly similar
			CT-P6				
			US-Herceptin®				
	IEC-HPLC	% Peak 5	EU-Herceptin®	63.2 – 72.1	66.4 ± 2.60	Highly similar	Highly similar
			CT-P6	62.2 – 65.8	63.9 ± 1.13		
			US-Herceptin®	63.2 – 65.9	64.6 ± 1.00		
		% Peak 7	EU-Herceptin®	0.9 – 2.4	1.5 ± 0.39	Highly similar	Highly similar
			CT-P6	2.9 – 4.0	3.6 ± 0.27		
			US-Herceptin®	0.9 – 1.5	1.2 ± 0.22		
Glycosylation	Galactosylated glycan by HILIC-UPLC	%[G1F-GN]+G1F+G2F	EU-Herceptin®	19.3 – 47.8	35.3 ± 9.52	Highly similar	Highly similar
			CT-P6	44.1 – 47.9	46.2 ± 1.15		
			US-Herceptin®	22.3 – 49.3	33.9 ± 10.45		
	Galactosylated glycan by LC-MS	%G1F+G2F	EU-Herceptin®	18.4 – 45.1	34.4 ± 8.21	Highly similar	Highly similar
			CT-P6	42.1 – 47.1	44.7 ± 1.23		
			US-Herceptin®	20.1 – 45.5	33.0 ± 10.04		
	Sialic acids by HILIC-UPLC	%[G1F-GN+NANA]+[G1F+NANA]+[G2F+NANA]+[G2F+2NANA]	EU-Herceptin®	0.3 – 2.2	1.2 ± 0.55	CT-P6 contains higher sialic acids compared to Herceptin®	CT-P6 contains higher sialic acids compared to Herceptin®
			CT-P6	2.1 – 3.3	2.9 ± 0.36		
			US-Herceptin®	0.5 – 1.7	1.1 ± 0.41		
	Sialic acids Analysis	NANA(sialic acid / protein, mol / mol)	EU-Herceptin®	0.03 – 0.06	0.05 ± 0.010	CT-P6 contains higher NANA compared to Herceptin®	CT-P6 contains higher NANA compared to Herceptin®
			CT-P6	0.12 – 0.14	0.13 ± 0.004		
			US-Herceptin®	0.03 – 0.06	0.04 ± 0.010		
	Glycation by intact mass	% Glycation at light chain	EU-Herceptin®	2.0 – 2.5	2.3 ± 0.16	Highly similar	Highly similar
			CT-P6	2.3 – 2.7	2.6 ± 0.10		
			US-Herceptin®	2.0 – 2.5	2.3 ± 0.15		
		% Glycation at heavy chain	EU-Herceptin®	3.1 – 3.7	3.4 ± 0.20	Highly similar	Highly similar
			CT-P6	4.0 – 4.7	4.4 ± 0.18		
			US-Herceptin®	2.8 – 3.8	3.4 ± 0.39		

1 In the Tier 2 assays, the QR limits were set based on the range of the values obtained for the reference product variation, expressed as 3 times Standard Deviation (SD). High similarity was considered to have been demonstrated if 90% of data points were within the QR of Herceptin® lots.

With regard to the biological quality attributes, most of the biological activity assays that do not directly assess mechanisms of action of trastuzumab were classified as Tier 2. A total of 8 assays to analyze F(ab′) and Fc related functionalities were evaluated by Tier 2.

**Figure 2.**
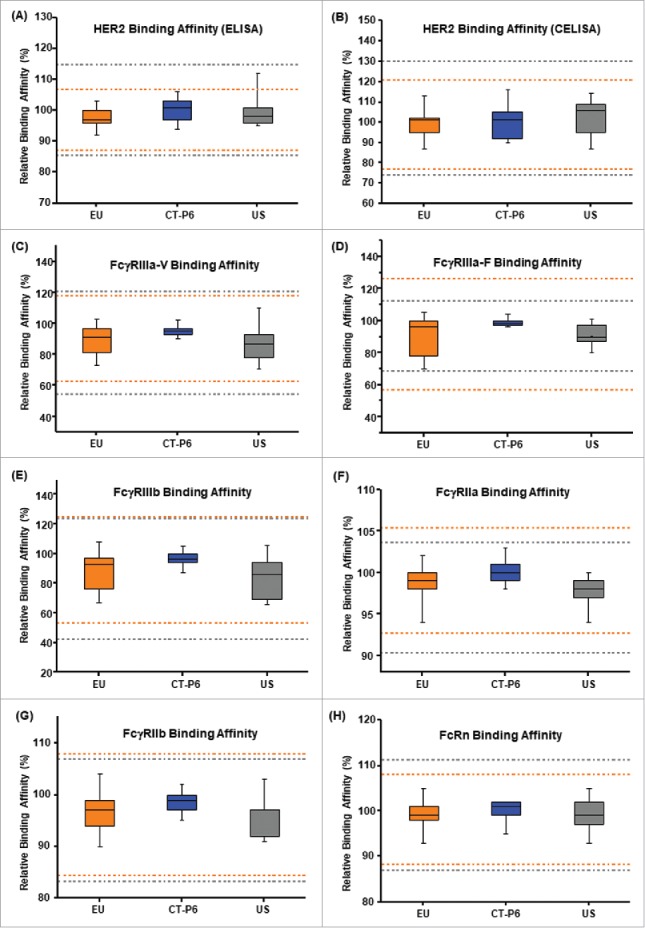
Comparison of Tier 2 biological attributes for CT-P6 (blue), EU-Herceptin® (orange) and US-Herceptin® (grey). Box plots of relative (A) HER2 binding affinity by ELISA, (B) cell-based HER2 binding affinity by CELISA, (C) FcγRIIIa-V binding affinity by SPR, (D) FcγRIIIa-F binding affinity by SPR, (E) FcγRIIIb binding affinity by SPR , (F) FcγRIIa binding affinity by SPR, (G) FcγRIIb binding affinity by SPR and (H) FcRn binding affinity SPR. Orange and grey broken lines represent quality range of EU-Herceptin® and US-Herceptin®, respectively. Box plot shows the interquartile range (box), median (band inside of box), maximum and minimum values (whiskers).

To evaluate F(ab′) related activities, HER2 binding affinity was measured by enzyme linked immunosorbent assay (ELISA) and cell-based ELISA (CELISA) methods. Although HER2 binding was ranked “very high” because trastuzumab mediates its activities through interaction with HER2,^36–41^ HER2 binding assays by either ELISA or CELISA were classified as Tier 2 since they do not directly measure clinically relevant mechanisms of action of the product. Tier 2 analysis showed that all values of HER2 binding affinity for CT-P6 were within the quality range of EU-Herceptin® and US-Herceptin® (Table 3, Fig. 2A and Fig. 2B). These results suggest that CT-P6 exhibits highly similar F(ab′) related activities compared with the reference products.

For Fc-related activities, relative binding affinity to FcγRIIIa-V, FcγRIIIa-F, FcγRIIIb, FcγRIIa, FcγRIIb and FcRn were measured by surface plasmon resonance (SPR). MAbs bound to a cell-surface antigen interact with FcγRs expressed on effector cells such as NK cells, neutrophils and macrophages, inducing these cells to exert cytotoxicity.^42^ Among the human Fcγ receptors (FcγRs), FcγRIIIa is well known as the only activating FcγR expressed on NK cells, and can play a pivotal role in ADCC induced by IgG1 subclass mAbs.^34–43^ FcγRIIIa is also found on the surface of resident monocytes, monocyte-derived dendritic cells and macrophages.^44^ FcγRIIa receptor has been reported to play a dominant role in the antibody-dependent cellular phagocytosis activity of macrophages.^45^^,^^46^ The binding to FcRn is considered an important quality attribute related to the PK profile.^47^ Based on this prior knowledge from literature, similarity data of relative binding affinities to FcγRIIIa-V, FcγRIIIa-F, FcγRIIIb, FcγRIIa, FcγRIIb and FcRn were analyzed by a Tier 2 quality range approach. Tier 2 analysis showed that all values of binding affinities to Fcγ receptors and FcRn for CT-P6 were well within the quality range of EU-Herceptin® and US-Herceptin® (Table 3, Fig. 2C to Fig. 2H). These results demonstrate that CT-P6 exhibits similar Fc functionality compared with EU- and US-Herceptin®.

With regard to the physicochemical properties, selected physicochemical attributes that are quantifiable and have been known to potentially affect clinical efficacy and safety were ranked as Tier 2. The various assays analyzed for primary structure, post-translational modification, higher order structure, purity/impurity, charge variants and glycosylation were included in Tier 2 analysis (Table 2). The statistical analysis using the quality range of the reference product revealed that, for most of Tier 2 attributes, more than 90% of data points were within the quality range of EU- and US-Herceptin®, as shown in Table 4.

A number of techniques were employed to compare the primary structure of CT-P6, EU- and US-Herceptin®, including molar absorptivity, peptide mapping (HPLC and LC-MS) and N/C-terminal sequencing. Although the risk ranking of primary structure is “high”, most of methods are qualitative, and thus not amenable to quantitative evaluation. Molar absorptivity and extinction coefficient provide quantitative data, so they were analyzed by quality range analysis. It was found that the molar absorptivity and extinction coefficient values of CT-P6 determined by measuring the optical density at a wavelength of 280 nm and protein molarity derived from the amino acid analysis were highly similar with EU- and US-Herceptin® (Table 4). The Tier 2 statistical analysis showed that 100% of the data points of molar absorptivity and extinction coefficient of CT-P6 were within the quality range of EU-Herceptin® as well as US-Herceptin® (Table 4).

**Figure 3.**
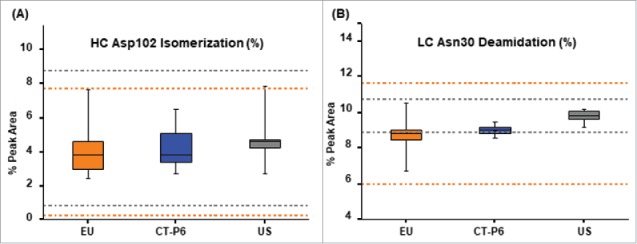
Box plots of (A) HC isoAsp102 and (B) deamidated LC Asn30 levels for CT-P6 (blue), EU-Herceptin® (orange) and USHerceptin® (grey). Orange and grey broken lines represent quality range of EU-Herceptin® and US-Herceptin®, respectively. Box plot shows the interquartile range (box), median (band inside of box), maximum and minimum values (whiskers).

Peptide mapping by LC-MS was used to check primary structure of the sample and to identify the post-translational modifications (deamidation, oxidation, isomerization, N-terminal pyroglutamate variants, C-terminal lysine variants and C-terminal proline amidation) of CT-P6, EU- and US-Herceptin®. Among the possible modifications, isomerization at HC Asp102 (HC isoAsp102) and deamidation at light chain (LC) Asn30 were considered to potentially impact product potency because they are located in the complementarity-determining region-3.^48^ In particular, isomerization at HC Asp102 has been known to severely reduce *in-vitro* anti-proliferation activity of trastuzumab.^49^ Tier 2 statistical analysis showed that levels of HC isoAsp102 and deamidated LC Asn30 in CT-P6 were within the quality range of EU-Herceptin® (Table 4), whereas, only 55.6% of data points for deamidated LC Asn30 in CT-P6 were within the quality range of US-Herceptin® (Table 4). This is due to the slightly lower deamidation level at LC Asn30 in CT-P6 compared to US-Herceptin®; therefore, no adverse impact is expected due to the difference. Box plots of these modifications for the comparison between CT-P6, EU- and US-Herceptin® are shown in Fig. 3A and Fig. 3B.

In the higher order structure characterization, free thiol levels determined by Ellman assay and thermal transition temperatures measured by differential scanning calorimetry (DSC) were evaluated using quality range analysis. HCs and LCs of mAbs are linked by disulfide bonds, which contribute to structural stability, thus free thiol content can reflect structural integrity of the product. The quality range evaluation suggested that 100% and 72.2% of CT-P6 lots were within the quality range (QR) of EU- and US-Herceptin®, respectively (Table 4). The 72.2% QR value of CT-P6 against US-Herceptin® originated from lower free thiol content of CT-P6, indicating slightly better structural integrity of CT-P6. DSC measures the heat capacity required to induce a change in the structure of a molecule, thus comparison of thermal transition temperatures is useful in comparing the higher order structure of the products. The Tier 2 statistical analysis showed more than 90% QR values in the three thermal transition temperatures corresponding to CH2, Fab, and CH3 unfolding. These results indicate that thermal stability and conformation of CT-P6 are highly similar to EU- and US-Herceptin® (Table 4).

**Figure 4.**
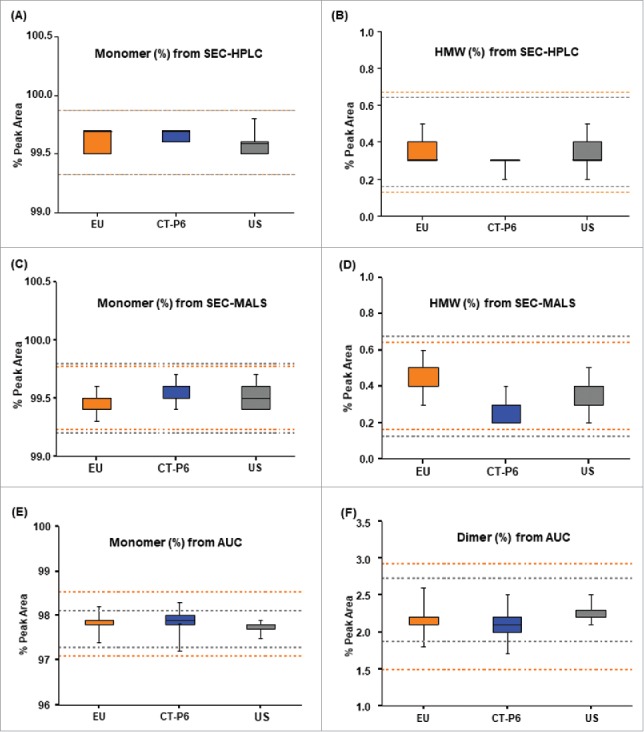
Comparison of monomer and aggregate contents for CT-P6 (blue), EU-Herceptin® (orange) and US-Herceptin® (grey). Box plots of (A) monomer % measured by SEC-HPLC, (B) HMW % measured by SEC-HPLC, (C) monomer % measured by SEC-MALS, (D) HMW % measured by SEC-MALS, (E) monomer % measured by AUC and (F) dimer % measured by AUC. Orange and grey broken lines represent quality range of EU-Herceptin® and US-Herceptin®, respectively. Box plot shows the interquartile range (box), median (band inside of box), maximum and minimum values (whiskers).

A wide range of methods were used to characterize the purity and impurity levels of the products (Table 2). Aggregation is a significant concern for biopharmaceutical products because it may be associated with decreased bioactivity and increased immunogenicity.^50–54^ Thus, the monomer and aggregate contents in CT-P6 and the reference products were thoroughly examined by three orthogonal methods, size-exclusion chromatography-high performance liquid chromatography (SEC-HPLC), SEC-multi-angle light scattering (SEC-MALS) and analytical ultracentrifugation (AUC). The QR evaluation and box plots for three methods are shown in Table 4 and Fig. 4, respectively. Tier 2 statistical analysis revealed that levels of monomer and high molecular weight (HMW) species in CT-P6 were highly similar (100% QR) with those in EU- and US-Herceptin® when analyzed by SEC-HPLC and SEC-MALS, whilst monomer and dimer levels obtained from AUC showed 88.9% QR. The low QR values in monomer and dimer levels in AUC were due to the slightly lower dimer content of CT-P6 than the reference products, indicating subtle improvement in aggregate content in CT-P6. Considering totality of the data from the three orthogonal methods, it can be concluded that there is no significant difference with respect to monomer and aggregate contents in CT-P6, EU- and US-Herceptin®.

**Figure 5.**
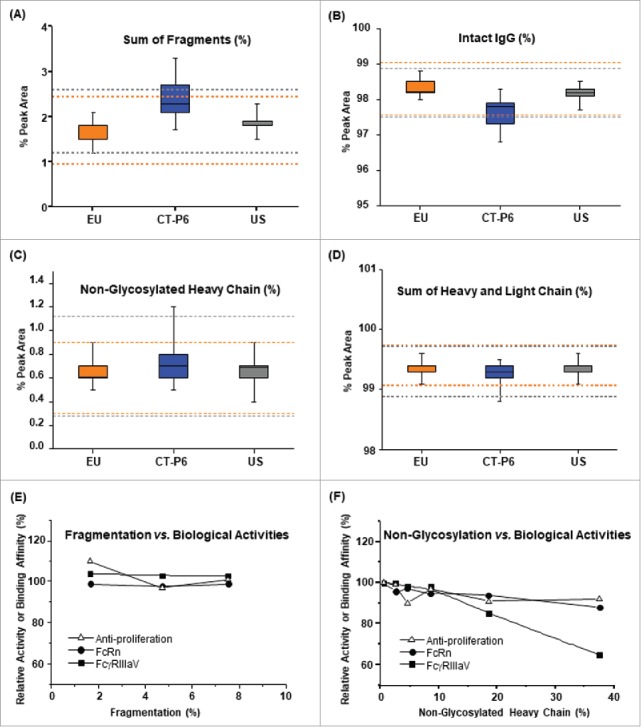
Comparison of fragmentation and non-glycosylation levels determined by CE-SDS for CT-P6 (blue), EU-Herceptin® (orange) and US-Herceptin® (grey). Box plots of (A) sum of fragments % by non-reduced CE-SDS, (B) intact IgG % by non-reduced by CE-SDS, (C) non-glycosylated heavy chain % by reduced CE-SDS and (D) sum of light and heavy chain % by reduced CE-SDS. Orange and grey broken lines represent quality range of EU-Herceptin® and US-Herceptin®, respectively. Box plot shows the interquartile range (box), median (band inside of box), maximum and minimum values (whiskers). Effects of fragmentation and non-glycosylation level on biological activities were examined in (E) and (F), respectively.

Fragmentation of mAbs is another source of product-related impurities that can potentially affect clinical efficacy, safety and PK profile because the presence of fragments may change the efficacy, reduced half-life, different biodistribution and safety profile of the product.^55^ Non-reduced CE-SDS was utilized to determine the level of fragmented species. As shown in Table 4 and Fig. 5A, the level of fragments in CT-P6 (2.3 ± 0.40%) was found to be moderately higher than that in EU-Herceptin® (1.7 ± 0.25%) and US-Herceptin® (1.9 ± 0.25%). The quality range analysis also showed lower than 90% QR values in the fragmentation level. The higher fragment content in CT-P6 leads to somewhat lower intact IgG level compared to the reference products (Fig. 5B). To evaluate the impact of fragmentation level on biological activities, anti-proliferation, ADCC, binding affinities to FcRn and FcγRIIIa-V were measured for CT-P6 samples containing various levels of fragmentation (Fig. 5E). The study results showed that up to approximately 5% of fragmentation level did not cause significant impact on the biological activities. Therefore, slightly higher fragmentation level in CT-P6 is not anticipated to impact on clinical efficacy of the product.

**Figure 6.**
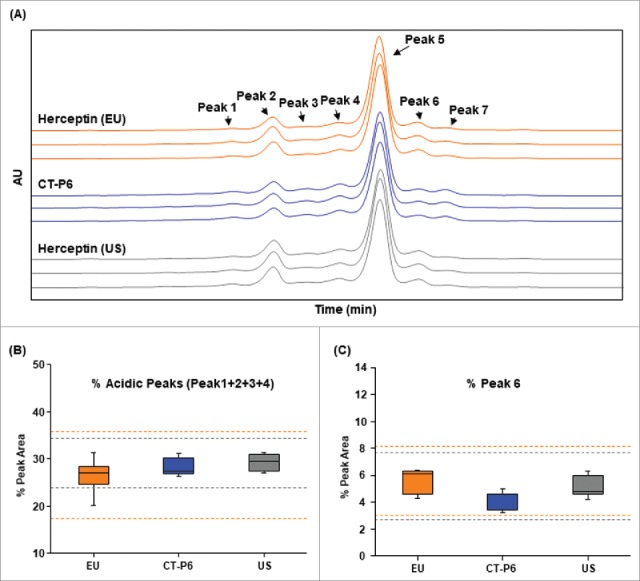
Comparison of charge variants of CT-P6 (blue), EU-Herceptin® (orange) and US-Herceptin® (grey) analyzed by IEC-HPLC. (A) Representative ion exchange chromatograms are presented for 3 batches of each product. The number and distribution of IEC-HPLC peaks are conserved between CT-P6 and RMPs. (B) Box plots of acidic peaks % (Peak 1 + Peak 2 + Peak 3 + Peak 4) in IEC-HPLC, (C) Box plot of Peak 6 % in IEC-HPLC. Orange and grey broken lines represent quality range of EU-Herceptin® and US-Herceptin®, respectively. Box plot shows the interquartile range (box), median (band inside of box), maximum and minimum values (whiskers).

The charge variants of mAbs are generated by common modifications such as oxidation, deamidation, isomerization, amination, cyclization, glycation, and the presence of C-terminal lysines.^56^ Depending on the locations and types of modification, charged isoforms may adversely impact on biological activities thus it is important to identify these charge variants in the product. The charge variant profile of CT-P6 consists of multiple molecular variants such as charged glycans, lysine variants, deamidated forms and isomerized forms. The charged isoforms are resolved to 7 peaks using ion exchange chromatography (IEC)-HPLC as shown in Fig. 6A. CT-P6, EU- and US-Herceptin® exhibited similar IEC-HPLC peak distribution. A peak fractionation study identified that Peak 6 contains HC isoAsp102 modification, therefore exhibits substantially reduced anti-proliferation activity. The study also showed that acidic peaks (Peak1, 2, 3 and 4) contain relatively higher levels of deamidated LC Asn30. Therefore, Peak 6 and sum of acidic peaks in IEC-HPLC were evaluated by Tier 2 statistical analysis. As shown in Fig. 6B and Fig. 6C, all data points of CT-P6 were within the quality range of EU- and US-Herceptin® with regard to the level of Peak 6 and acidic peaks. These results suggest that the content of charge variants in CT-P6 is highly similar compared with that in the reference products.

Glycosylation of mAbs plays an important role for complement-dependent cytotoxicity (CDC) and ADCC function by modulating the binding to the Fcγ receptor,^57^^,^^58^ and affects the antibody conformation and thermal stability.^59^ Therefore, it is important to carefully establish risk ranking related to the glycosylation. Due to the complexity of the structures and the variety of effects on the product, the glycoforms were assessed in five different glycan groups: 1) non-glycosylated product, 2) afucosylated glycans, 3) mannosylated glycans, 4) galactosylated glycans, and 5) sialylated glycans. Among these glycoforms, non-glycosylation, afucosylation and mannosylation were classified as Tier 2.

Non-glycosylation may critically impact product potency and clinical efficacy considering the crucial roles of glycosylation on a mAb's function. We have determined the level of non-glycosylation of trastuzumab by reduced CE-SDS. The similarity assessment by reduced CE-SDS showed that the level of non-glycosylated HC in CT-P6 (0.8 ± 0.20%) was slightly higher than that in EU-Herceptin® (0.6 ± 0.10%) and US-Herceptin® (0.7 ± 0.14%) (Table 4 and Fig. 5C). The Tier 2 statistical analysis also revealed 77.8% QR and 88.9% QR when EU- and US-Herceptin® quality ranges were applied, respectively. The slightly higher non-glycosylation in CT-P6 leads to the slightly lower level of HC and LC sum as shown in Fig. 5D. Nevertheless, a spiking study using fully aglycosylated CT-P6 sample showed that there is no adverse impact on various biological activities up to about 3% of non-glycosylation (Fig. 5F). These results suggest that a slightly higher level of non-glycosylated HC in CT-P6 is highly unlikely to have any clinical impact.

**Figure 7.**
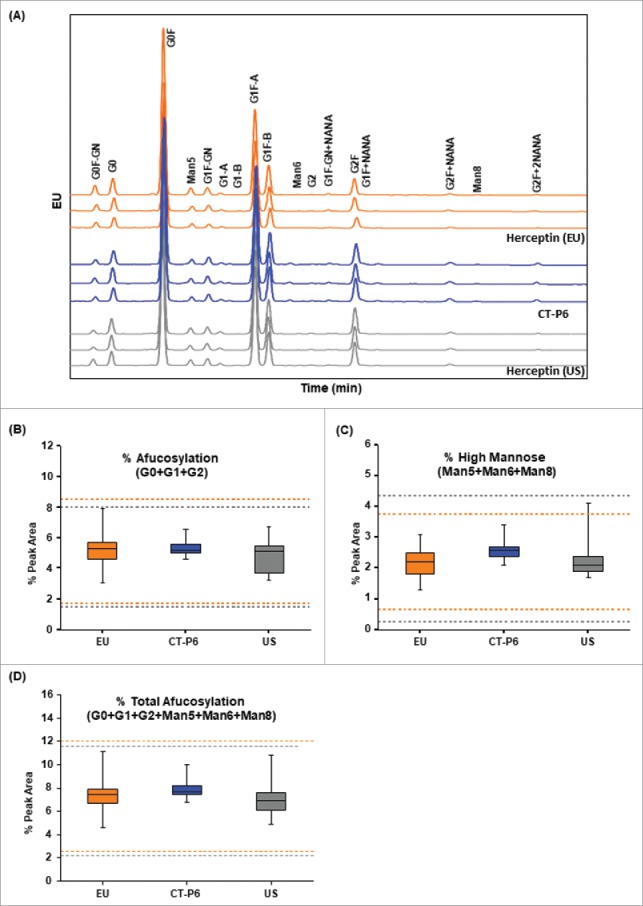
Comparison of oligosaccharide profiles of CT-P6 (blue), EU-Herceptin® (orange) and US-Herceptin® (grey) analyzed by HILIC-UPLC with 2-AB labeling. (A) Representative oligosaccharide profiles are presented for 3 batches of each product. The types and proportions of the glycans are conserved among the products. (B) Box plot of afucosylated glycans % (G0+G1+G2), (C) Box plot of high mannose glycans % (Man5+Man6+Man8), (D) Box plot of total afucosylated glycans % (G0+G1+G2+Man5+Man6+Man8). Orange and grey broken lines represent quality range of EU-Herceptin® and US-Herceptin®, respectively. Box plot shows the interquartile range (box), median (band inside of box), maximum and minimum values (whiskers).

To determine the relative content of afucosylated and mannosylated glycans, oligosaccharide profiling by 2-aminobenzamide (2-AB)-labeled hydrophilic interaction liquid chromatography-ultra-performance liquid chromatography (HILIC-UPLC) was performed. The oligosaccharide profile showed that the types and proportions of the glycans were reasonably conserved between CT-P6 and the reference products (Fig. 7A). With regard to afucosylated glycans (e.g., G0, G1 and G2), an extensive body of literature has shown that IgG1 lacking core fucose has increased binding affinity for FcγRIIIa, and subsequently exhibits enhanced ADCC activity.^60–64^ A in-house spiking study using highly afucosylated CT-P6 also demonstrated strong positive correlation of afucosylation level with FcγRIIIa binding affinity, as well as ADCC activity (data not shown). Mannosylated glycans do not contain fucose; therefore, it has been reported that high mannose glycoforms exhibit higher FcγRIIIa binding and ADCC activity.^65–67^ However, it has been shown that mannosylated glycans induce fast clearance of IgG1 via a mannose receptor mediated mechanism.^68^^,^^69^ The similarity assessment by oligosaccharide profiling showed that the level of afucosylation and high mannose in CT-P6 were similar with ones observed in EU- and US-Herceptin® (Table 4, Fig. 7B and Fig. 7C). Total afucosylation level was also found to be highly similar between the products (Fig. 7D). The Tier 2 statistical analysis revealed all data points in CT-P6 were within the quality range of EU- and US-Herceptin® for afucosylation, high mannose and total afucosylation levels (Table 4). Consistent results were obtained by N-linked glycan analysis by LC-MS (Table 4). Taken together, it was concluded that CT-P6 contains highly similar afucosylated and mannosylated glycans compared with EU- and US-Herceptin®.

### Similarity evaluation by Tier 3

Tier 3, evaluation through visual comparison, is used for attributes with lowest risk ranking and test of qualitative results. Summary of analytical results and similarity evaluation for Tier 3 attributes are shown in Table 3 and Table 4.

In the biological activity assays, C1q binding affinity by ELISA and FcγRI binding affinity by SPR were ranked as Tier 3 due to their low risk ranking scoring. Table 3 showed that C1q binding affinity and FcγRI binding affinity of CT-P6 were highly similar compared with EU- and US-Herceptin®.

**Figure 8.**
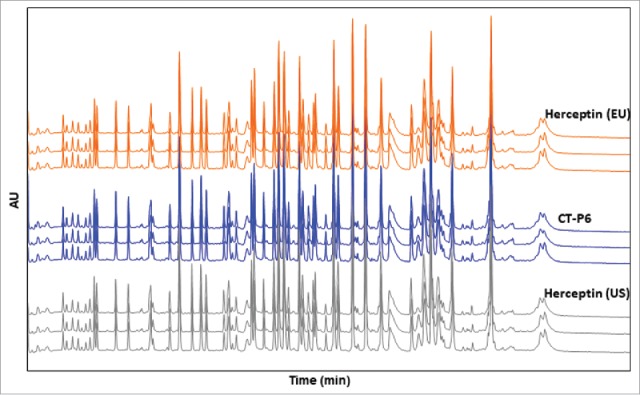
Comparison of UV chromatograms of trypsin digested CT-P6 (blue), EU-Herceptin® (orange) and US-Herceptin® (grey). Representative tryptic peptide maps detected at 214 nm are presented for 3 batches of each product.

In the physicochemical tests, a number of qualitative methods were evaluated through visual comparison. For primary structure characterization, peptide mapping by HPLC and LC-MS were conducted. UV-based tryptic peptide mapping revealed a highly similar peak profile among the products without missing or additional new peaks, and with comparative retention time of each peak (Fig. 8). Total ion chromatograms of the tryptic peptide map obtained by LC-MS were also highly similar between CT-P6 and the reference products (data not shown). N- and C-terminal sequence determined by peptide mapping LC-MS were found to be same between the products. These data support the conclusion that primary structure of CT-P6 is identical with EU- and US-Herceptin®.

Among the post-translational modifications analyzed by peptide mapping LC-MS, oxidation at HC Met255, N-terminal pyroglutamate at HC Glu01, C-terminal clipped lysine and C-terminal proline amidation at HC were ranked Tier 3, considering their relatively low risk ranking. The results showed that all above modification levels were highly similar between CT-P6 and the reference products except for C-terminal proline amidation (Table 4). C-terminal proline amidation was found to be higher in CT-P6 compared to that in the reference products, with a range of 2.2 – 3.1% for CT-P6 and 0.1 – 0.4% for the reference products. The proline amidation at C-terminal is located away from functionally related (Fc or Fab) regions. Also, C-terminal proline amidation has been reported to have no impact on Fc effector function, Fab binding activity and the product safety of an IgG1 antibody.^70^^,^^71^ Overall, the three products showed highly similar biological activities, suggesting that the observed difference in C-terminal proline amidation has no impact on efficacy *in vivo*.

**Figure 9.**
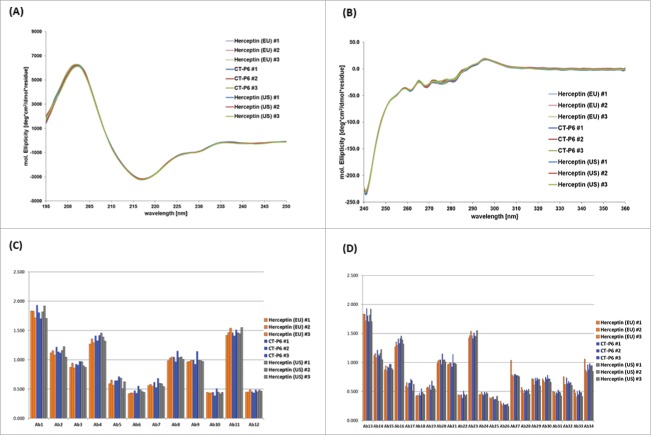
Higher order structure analyzed by CD and antibody conformational array for CT-P6, EU-Herceptin® and US-Herceptin®. CD spectra for (A) Far-UV region and (B) Near-UV region are overlaid. Histograms of epitope exposure analyzed by antibody conformational array in (C) variable region, and (D) constant region are compared for 3 batches of each product.

For higher order structure characterization, various qualitative methods were utilized. Disulfide bond positions by means of native and reduced peptide mapping coupled with LC-MS revealed eight new peaks in the native peptide map that were identified as disulfide bond linked peptide based on the MS and MS/MS sequence analysis. These eight disulfide bond linked peptides were matched in all products, indicating the presence of the same inter- and intra-chain disulfide bond linages in all products. The secondary or tertiary structures of the molecule were analyzed by circular dichroism (CD) and Fourier transform infrared spectroscopy (FT-IR). The far-UV and near-UV CD spectra of CT-P6, EU- and US-Herceptin® showed the typical shape of an antibody with highly similar spectral signal and mean residue molar ellipticity (MRE) (Fig. 9A and Fig. 9B). In FT-IR spectra, all samples agreed well with respect to shape and location of the amide I range of 1,641 to 1,642 cm^−1^, the amide II band at the range of 1,553 to 1,554 cm^−1^ and the 3 characteristic bands between 1,200 and 1,500 cm^−1^ (data not shown). Antibody conformational array of the three products showed highly similar epitope exposure in variable and constant regions against the 34 pools of polyclonal antibodies (Fig. 9C and Fig. 9D). These data indicated that the three products have highly similar secondary and tertiary structures.

The levels of process-related impurities, including residual host cell protein, host cell DNA, residual rProtein A and sub-visible particles, were found to be highly similar between CT-P6 and the reference products. Charge variants analyzed by isoelectric focusing (IEF) and IEC-HPLC exhibited highly similar results between the products.

With regard to the glycosylation, galactosylated glycans, sialic acid and glycations were compared between CT-P6 and the reference products. Galactosylated glycans have been known to affect CDC activity,^72^^,^^73^ but their risk ranking was determined to be low because CDC is not a mechanism of action by which trastuzumab exerts its therapeutic effect.^74^ The oligosaccharide profiling results showed CT-P6 has a higher mean value of galactosylated glycan level (46.2 ± 1.15%) than EU-Herceptin® (35.3 ± 9.52%) and US-Herceptin® (33.9 ± 10.45%) (Table 4). Nevertheless, it should be noted that EU- and US-Herceptin® exhibit a wide range of galactosylation levels; thus, all CT-P6 lots remained within the minimum and maximum values observed in EU- and US-Herceptin®. Chinese hamster ovary (CHO) cell-expressed IgG is known to contain only trace amount of N-acetylneuraminic acid (NANA), which is not immunogenic.^75^ We consistently found that very low levels of NANA were found in CT-P6 and Herceptin® that are expressed in the CHO cell line. Oligosaccharide profiling and sialic acid analysis showed that NANA levels in CT-P6 were higher than those of EU- and US-Herceptin®. However, the marginally higher amount of NANA present in CT-P6 is not expected to cause adverse effects because the absolute amount of NANA present in CT-P6 remains very small and NANA is a non-immunogenic glycan species. Glycation is a post-translational chemical reaction that results in non-enzymatic addition of glucose to protein amine groups, primarily the alpha amino terminal and epsilon amino group on the lysine side chain.^76^ The impact that glycation has on therapeutic antibodies is somewhat controversial due to the fact that the effect of glycation is protein specific, depending on exposed lysine residues. Glycation levels at LC and HC determined by intact mass showed that CT-P6 contains similarly low levels of glycation compared with EU- and US-Herceptin® (Table 4).

## Discussion

CT-P6 is being developed by Celltrion Inc. as a biosimilar product to the original trastuzumab, Herceptin®. A comprehensive analytical similarity assessment was performed using a wide range of sensitive, orthogonal and state-of-the-art methodologies to demonstrate similarity of CT-P6 to the reference product Herceptin® obtained from the two different markets, EU and US. The similarity assessment was performed to characterize primary structure, higher order structure, purity/impurity, charge isoforms, glycosylation and biological activities of trastuzumab.

The analytical data from the similarity study was evaluated using the statistical analyses recommended by FDA in its recent draft guideline. In this risk-based approach, various quality attributes of products are first ranked according to their risk of potential clinical impact. These attributes or assays are then evaluated by one of three tiers of statistical analysis based on a risk ranking, as well as other factors. Evaluation of analytical similarity has been a challenging issue for the biosimilar industry because the availability of lots for reference product and biosimilar product are limited at the time of development, while the measurable quality attributes of target molecule are numerous, which can lead to potential bias or false negative/positive conclusions about biosimilarity. Therefore, the risk-based tier approach provides a systematic pathway to evaluate analytical similarity with a high level of confidence and lower potential bias.

The data from our extensive similarity exercise successfully demonstrated that CT-P6 is highly similar to EU-Herceptin® and US-Herceptin®. Similarity of anti-proliferation and ADCC, the two biological activities with highest potential clinical impact, were evaluated by the equivalent test (Tier 1). The results demonstrated that 90% CI of mean difference between CT-P6 and the reference product is within the equivalence margin in the anti-proliferation and ADCC assays. Most of the other biological activities related to F(ab′) or Fc functions were evaluated by the quality range approach (Tier 2). The measured binding affinities of all CT-P6 lots were within the quality range of EU- and US-Herceptin®. These results strongly suggest that CT-P6 exhibits highly similar functional properties compared with the reference products. Similarity of the structural and physicochemical properties in trastuzumab were evaluated by either Tier 2 or visual comparison (Tier 3) depending on their risk ranking. Tier 2 and Tier 3 analyses showed highly similar primary/higher order structures, purity/impurity profiles, charge isoforms and glycosylation between CT-P6 and the reference products. In the Tier 2 analysis, more than 90% of CT-P6 lots compared were within the quality range of the reference product for most quality attributes. Although slightly higher levels of fragmentation and non-glycosylation were observed in CT-P6, the differences were very small and unlikely to be clinically meaningful. The attributes with low risk ranking or qualitative assay examined by visual comparison also showed highly similar results between CT-P6 and the reference product. These results support that CT-P6 has highly similar structural/physicochemical properties compared with the reference products. A recently reported clinical outcome study further supports comparable efficacy and safety of CT-P6 with Herceptin®.^77^^,^^78^ Taken together, it can be confidently concluded that CT-P6 is a highly similar product compared with the reference product in view of the totality of the evidence.

## Materials and methods

### Materials

CT-P6 were manufactured at Celltrion Inc., Korea. Herceptin® lots were purchased from a pharmacies located in the EU and US.

### Tier 1 statistical analysis

Tier 1 statistical analysis uses the equivalence test with the null hypothesis;

· H_0_: $\mu_{T}$ – $\mu_{R}$ ≤ - δ or $\mu_{T}$ – $\mu_{R}$≥ δ
· H_1_: - δ < $\mu_{T}$ – $\mu_{R}$ < δ

Where $\mu_{T}$ stands for mean of tested product; $\mu_{R}$ stands for mean of the reference product; and δ stands for equivalence margin (EM) based on the reference product variability (standard deviation). The EM for demonstrating similarity was defined as ± 1.5*Standard Deviation (SD) based on the reference product. The value of 1.5 was established by the FDA.^25^

The confidence interval (CI) was used to determine whether the mean difference for functional biological measures from T (test or proposed biosimilar) and R (reference) products are similar or not. Prior to calculating the CI to assess similarity, homoscedasticity among products was checked using an F-test, which is used to test if the variances of products are equal. The p-value gives the probability of a greater F value under the null hypothesis that population variances are equal against the alternative that the two are different. F-test statistics is:

$$\text{F}=\frac{MAX\left({\hat{\sigma }}_{T}^{2},{\hat{\sigma }}_{R}^{2}\right)}{MIN\left({\hat{\sigma }}_{T}^{2},{\hat{\sigma }}_{\text{R}}^{2}\right)}$$

Where $n_{T}$ stands for number of test products; $n_{R}$ stands for number of the reference products;$\;{\hat{\sigma }}_{T}^{2}$ stands for variance of test products;$\;{\hat{\sigma }}_{R}^{2}$ stands for variance of the reference products the null hypothesis is rejected if F > $F_{\alpha /2,df1,df2}$ (where $F_{\alpha /2,df1,df2}$ is upper tail critical value for the $F_{df1,df2}$ distribution). If ${\hat{\sigma }}_{T}^{2}>{\hat{\sigma }}_{R}^{2}$ then ${df1=n}_{T}-1\;and\;df2=n_{R}-1\;.\;\;if\;{\hat{\sigma }}_{T}^{2}<{\hat{\sigma }}_{R}^{2}$ then $df1=n_{R}-1\;and\;df2=n_{T}-1.$

If the result of the F-test is not significant, implying equal variance, the 90% confidence interval on the difference is calculated using pooled standard deviation as below.

· (100(1-2α))% Confidence Interval of Mean Difference
  $$({\hat{\mu }}_{T}-{\hat{\mu }}_{R}{}_{})\pm t_{1-\alpha ,df^{*}}\sqrt{{\hat{\sigma }}_{p}^{2}\left(\frac{1}{n_{T}}+\frac{1}{n_{R}}\right)}$$

Where ${\hat{\sigma }}_{p}^{2}=\;\frac{(n_{T}-1){\hat{\sigma }}_{T}^{2}+(n_{R}-1){\hat{\sigma }}_{R}^{2}}{n_{T}+n_{R}-2}$ and $df^{*}=n_{T}+n_{R}-2$

If the result of the F-test is significant, implying unequal variance, 90% confidence interval on the difference is computed assuming the Satterthwaite method using the following formula

· (100(1-2α))% Confidence Interval of Mean Difference
  $$\left({\hat{\mu }}_{T}-{\hat{\mu }}_{R}\right)\pm t_{1-\alpha ,df*}\sqrt{\frac{{\hat{\sigma }}_{T}^{2}}{n_{T}}+\frac{{\hat{\sigma }}_{R}^{2}}{n_{R}}\;}df*=\frac{{\left(\frac{{\hat{\sigma }}_{T}^{2}}{n_{T}}+\frac{{\hat{\sigma }}_{R}^{2}}{n_{R}}\right)}^{2}}{\frac{{\hat{\sigma }}_{T}^{4}}{n_{T}{}^{2}(n_{T}-1)}+\frac{{\hat{\sigma }}_{R}^{4}}{n_{R}{}^{2}(n_{R}-1)}}$$

Equivalence between 2 products was confirmed if 90% CI of mean difference was within the corresponding EM. Equivalence test was calculated by T-TEST PROCEDURE in SAS Enterprise Guide 7.1 (SAS Institute Inc., Cary, NC, USA).

### Tier 2 statistical analysis

Tier 2 statistical analysis uses the quality range of the reference product:

· $\left(\mu_{R}-\text{x}\sigma_{R},\;\mu_{R}+\text{x}\sigma_{R}\right)$

Where *μ_R_* stands mean of the reference product; σ_R_ stands for variation (standard deviation) of the reference product; and x stands for multiplicity of unit reference product variation (multiplier).

The quality range (QR) was set based on the range of the values obtained from the reference product variation expressed as x times the reference product standard deviation (±xSD). The multiplier x has been determined for each Tier 2 attribute based on the variability of each assay and the relative importance of the attribute to the safety and efficacy of the product. The multiplier 3 was used for all Tier 2 attributes. Based on FDA criteria, high similarity was considered to have been demonstrated if 90% of data points for the test product were within the QR of the reference products.^79^^,^^80^

### Tier 3 evaluation

The raw data/graphical data are presented to allow comparison of quality attributes with a low risk ranking or for qualitative test methods.

### Sample size calculation

The sample size for the similarity assessment was calculated based on assay variation. The variation of 6 batches each of US-Herceptin®, which were obtained from the initial similarity study, were evaluated with respect to biological activities representative of in vitro bioactivity and ADCC as Tier 1. The number of batches required to obtain an overall statistical power of 90% were calculated using an EM defined as ± 1.5 * Standard Deviation (SD of US-Herceptin®) as this is considered as the most conservative statistical approach for assessment of similarity. A statistical sample-size calculation program, PASS ver.13.0 was used.^81^ Following this approach, the largest sample size was calculated to be 11 samples for the assays. Therefore, more than 11 batches of each product were included in the similarity assessment. A total 18 batches of CT-P6, 17 batches of EU-Herceptin® and 12 batches of US-Herceptin® were used for the similarity assessment.

### Amino acid analysis and molar absorptivity

After buffer exchange with phosphate-buffered saline, all samples were hydrolyzed with 6 N HCl (including ∼1% phenol) under reduced pressure at 112°C for a 24 hrs. Decomposed amino acids were reconstituted and derivatized by pre-column derivatization method with 6-aminoquinolyl-N-hydroxysuccinimidyl carbamate (AQC reagent) so that the sample could be detected by fluorescence detector. Each amino acid was quantitated with internal standard method.

Molar absorptivity was determined according to Beer-Lambert Law. First, measurements of OD_280_ and OD_320_ were performed using a Beckman DU730 spectrophotometer. Then, protein concentration was obtained from the amino acid analysis using the concentration of ‘robust’ amino acids (where % deviation between observed and expected results was ≤ 5%) such as aspartic acid, glycine, arginine, alanine, proline, valine and leucine. Molar extinction coefficient was calculated with UV absorbance at 280 nm, concentration of protein and molecular weight of CT-P6 and Herceptin®.

### HPLC peptide mapping analysis

Samples were analyzed by HPLC peptide mapping after reduction with dithiothreitol (DTT) (Sigma-Aldrich), alkylation with iodoacetamide (IAM) (Sigma-Aldrich), and digestion with trypsin (Promega) at 37°C. The resulting peptides were separated by reversed phase-HPLC using a C_18_ column (5 µm, 250 × 4.6 mm; Vydac, Hesperia) and acetonitrile gradient (Burdick & Jackson) containing trifluoroacetic acid (Sigma-Aldrich). Absorbance was monitored at 214 and 280 nm using a Waters-2695 Alliance HPLC system equipped with a UV detector (Waters).

### LC-MS-ESI peptide mapping analysis

Samples were analysed by LC-MS peptide mapping after reduction with DTT, alkylation with IAM, desalting with NAP5 column and digestion with trypsin (a 20 (sample):1 (trypsin) treatment, at 37°C for 2 hrs), Asp-N (a 100 (sample):1 (Asp-N) treatment, at 37°C for 16 hrs). The resulting peptides were separated by reversed-phase UPLC using a C18 column and a gradient of acetonitrile containing formic acid. The chromatography was performed on a Waters Acquity UPLC. An online AB SCIEX Triple TOF 5600 mass spectrometer with an electrospray source was used to collect mass spectra of the intact peptide, as well as to fragment the peptides for sequencing (MS/MS analysis). The m/z (mass/charge) data were collected from 250 to 1,600 m/z.

### Free thiol analysis by Ellman assay

The free thiol (SH) groups in the samples were determined by means of the 5,5′-dithiobis-(2-nitrobenzoic acid) (DTNB, Ellman's reagent) method. Briefly, standard and samples were mixed with DTNB in 7.5 M guanidine HCl, 125 mM sodium phosphate pH 8.0 and 1.25 mM EDTA, followed by measurement of absorbance at 412 nm. Free thiol groups could be estimated in a sample by comparison to a standard curve composed of known concentrations of a sulfhydryl-containing compound such as cysteine. The results were reported as molar ratios (free SH/IgG, μM/μM).

### Disulfide bond analysis

Samples were analyzed by comparing native and reduced peptide maps. In the case of reduced peptide mapping analysis, the samples were reduced with DTT and alkylated with IAM; whereas in the case of native peptide mapping analysis, no DTT was added to the sample. The samples were digested using trypsin after desalting with NAP-5 column. The resulting peptides were separated by reversed-phase UPLC using a C18 column and a gradient of acetonitrile containing formic acid. The chromatography was performed on a Waters Acquity UPLC. An online AB SCIEX Triple TOF 5600 mass spectrometer with an electrospray source was used to collect mass spectra of the intact peptide, as well as to fragment the peptides for sequencing (MS/MS analysis).

### Fourier transform infra-red spectroscopy

FT-IR spectra were recorded on a Nicolet 6700 (Thermo-Nicolet) with a smart iTR ATR accessory and diamond crystal at a resolution of 4 cm^−1^, number of scans 32, and spectral range from 4000 – 650 cm^−1^. All spectra were baseline and ATR-corrected with instrument software. In order to generate the difference spectra, buffer spectra were recorded as a blank and subsequently subtracted. Sample solutions were transferred onto the crystal and allowed to dry. FT-IR spectra were analyzed by comparing the locations and shapes of the amide I and amide II bands, as well as three other bands between 1200 and 1800 cm^−1^.

### Circular dichroism spectroscopy

CD experiments were performed using a Chirascan-plus CD spectrometer (Applied Photophysics) equipped with a Pelltier controlled temperature regulator at 20°C. Cells of quartz glass along with optical path lengths of 1.0 and 0.10 cm were used for near-UV and far-UV CD measurements, respectively. Protein concentrations used for the far-UV spectra and near-UV CD spectra were 0.2 and 1.0 mg/ml, respectively. The formulation buffer was measured as a blank and subsequently subtracted. Noise reduction was applied to the baseline-corrected protein spectra using the smoothing option of the device software for the spectrometer. Conversion of the measured CD signals to mean residue molar ellipticities [(θ)_MRE_] was also performed using this software.

### Differential scanning calorimetry

Thermal stability of the samples was evaluated by measuring their T_m_ values using a Microcal VP-DSC microcalorimeter (MicroCal). The thermogram was obtained with a scan rate of 1°C/min within a temperature range of 30 to 100°C. DSC data were analyzed using a non-two state, 3 transition model to get the melting points of the transition temperatures using the Origin software package (OriginLab Corporation) to determine three thermal transition temperatures.

### Antibody array

CT-P6, EU-Herceptin® and US-Herceptin® samples were tested pairwise in triplicate using a HercBridge ELISA kit (ArrayBridge Inc., Cat. No. AB000207,). The assay was performed by making a 5 µg/ml solution of both CT-P6 and Herceptin® respectively, and adding to the 96-well plate. Following a 1 hour incubation to allow capture of the proteins by the panel of antibodies on the plate, a reporting polyclonal anti-human IgG antibody, conjugated with biotin, was added and incubated for 1 hour to allow it to bind to any captured proteins. After this incubation, the plate was washed and a streptavidin-horseradish peroxidase (HPR) conjugate was added and incubated for 45 minutes. The streptavidin-HRP conjugate was captured by any biotin labeled antibody bound to the plate. Following a wash step to remove unbound conjugate, 3, 3′, 5, 5′-tetramethylbenzidine (TMB) substrate was added and was converted by the captured HRP to a colored product in proportion to the amount of HRP bound to the plate. After a short incubation to allow color development, the reaction was stopped and the intensity of the generated color was detected in a microtiter plate reader capable of measuring 450 nm wavelength. The color development was proportional to the captured test mAb or the reference mAb. All test plates were handled identically, enabling side-by-side comparison of all samples.

### Size-exclusion chromatography

Samples were diluted to 5 mg/mL with mobile phase buffer (20 mM NaH_2_PO_4_, 150 mM NaCl, pH 7.0) prior to the analysis. SEC-HPLC was performed under non-denaturing conditions with the Waters-2695 Alliance HPLC system on a TSK G3000SW_XL_ column (Tosoh, Japan) with the aqueous-buffered mobile phase. The isocratic elution profile was monitored using UV detection at 214 nm.

### SEC-multi angle light scattering

SEC-MALS was performed by HPLC on a TSKgel G3000SWXL column using aqueous buffered mobile phase (20 mM NaH_2_PO_4_, 150 mM NaCl, pH 7.0). The isocratic elution profile was monitored using MALS system, DAWN® HEREOS™II and Optilab rEX (Wyatt Technologies). Molecular weight and the monomer and high molecular weight (HMW) content was determined with MALS and RI detector.

### Sedimentation velocity analytical ultracentrifugation

Sedimentation velocity analytical ultracentrifugation (SV-AUC) was carried out on a Beckman Coulter XLA-70 AUC instrument at 20°C. Sample was loaded into the sample channel of AUC cells having quartz windows and 12-mm double-sector Epon centerpieces. Matching buffer having the same composition and pH as the sample were loaded into the corresponding reference channel of each cell. The centrifugation was carried out at 20°C and 45,000 rpm. Radial scans of the concentration profile were collected sequentially by absorbance at 280 nm, until full sedimentation was reached. The resulting datasets were analyzed using the program SEDFIT with a continuous c(s) distribution model, yielding best-fit distributions for the number of sedimenting species and the effective molecular weights. Each sample was analyzed in two separate SV-AUC spins, resulting in two analyses per sample. The resulting c(s) distribution profile was used to calculate the percentage of each species and the estimated molecular weights.

### Capillary sodium dodecyl sulfate gel electrophoresis

CE-SDS was performed under both non-reducing conditions for analysis of purity/impurities. A Beckman Coulter, PA 800 capillary electrophoresis system was used with a 67 cm, 50 μm I.D. bare-fused silica capillary. Reduced CE-SDS was performed for determination of purity as the corrected peak area % of sum of HC and LC, and non-glycosylated HCs. The samples were reduced using 2-mercaptoethanol and subjected to electrophoresis under reducing condition. Non-reduced CE-SDS was performed for determination of the corrected peak area % of intact IgG and non-assembled IgG molecules. The samples for non-reduced CE-SDS were alkylated using 250 mM IAM and subjected to electrophoresis under non-reducing condition.

### Isoelectric focusing

IEF was used to determine pI values of charge variants in the samples. Electrophoresis was performed on IsoGel agarose IEF plates in the pH range of pH 3 – 10 using a flatbed electrophoresis system (Multiphor II, Amersham). pI values were calculated against IEF pI markers (pI range: 9.45 – 6.0) and compared to the pI values of the reference standard. The samples were focused by running the gels at 1500 V, 7 mA and 25 W for 75 minutes. Following focusing, the gels were stained with PhastGel Blue R, dried and scanned. pI values were calculated using Quantity One software (Bio-Rad).

### Ion exchange chromatography

The IEC-HPLC method was used to evaluate the distribution of charge variants by cation exchange chromatography. The HPLC system (Waters) was equipped with a Propac WCX 10 analytical column (4 × 250 mm) and guard column (4 × 50 mm) set (Dionex) at ambient temperature. Gradient NaCl elution was performed at a constant flow rate of 0.8 mL/min and UV signals were obtained at 214 nm. Peaks in the IEC HPLC chromatogram were integrated, and percentage peak areas of each peak were calculated.

### Oligosaccharide profile analysis by hydrophilic interaction liquid chromatography

For oligosaccharide profile analysis, N-linked glycans were released from the antibody using PNGase F treatment at 37°C. PNGase F-cleaved glycans were analyzed by UPLC with a FLD detector (Waters). Released N-linked glycans by PNGase F treatment were extracted from deglycosylated protein solution using a GlycoClean H Cartridges (Prozyme). Extracted glycans were labeled with 2-AB labeling reagent, followed by removal of excess labeling reagent using a GlycoClean S Cartridges (Prozyme). Finally, 2-AB-labeled N-linked glycans were analyzed by normal phase chromatography with a BEH glycan column (Waters) and fluorescence detector.

### *N*-glycan analysis by LC-MS

For structural analysis of *N*-linked oligosaccharide at Asn 300 by LC-MS (AB SCIEX), trypsin-digested peptides prepared for peptide mapping were evaluated. Extracted ion chromatograms were used to quantify each oligosaccharide species. The percentage calculation was based on each glycosylation site. For each site, all detectable oligosaccharide structures were counted.

### Sialic acid analysis

Sialic acids were released from the antibody by mild acid hydrolysis (0.1 M HCl) for 1 hr at 80°C. A Zorbax Extend-C18 column was used at a flow rate of 1.0mL/min and following gradient conditions using 7% methanol/ 93% water (mobile phase A) and 7% methanol/93% acetonitrile (mobile phase B); 5% B initially for 35 min, increased to 100% B in 0.1 min, followed by 9.9 min isocratic hold. Initial conditions were restored in 0.1 min and held for an additional 14.9 min to ensure column equilibration. The chromatography was performed on a Waters HPLC system with fluorescent detector (FLD). The sialic acid content was quantified based on the response of sialic acid standards (NANA) relative to an internal standard [2-keto-3-deoxy-D-glycero-D-galacto-nononic acid; deaminated neuraminic acid]. The results were reported as molar ratios (sialic acid/protein, mole/mole).

### Glycation analysis

Samples were digested with PNGase F (1,500 U/mg treatment, at 37°C for 16 hours) for removing N-glycan, and then reduced with DTT followed by LC-ES-MS analysis using an Agilent 1200 HPLC coupled online to an Agilent 6530 Q-TOF mass spectrometer. The m/z (mass/charge) data were collected from 900 to 4,000 m/z at a scan rate of 1 spectrum per second followed by deconvolution of the mass spectra to intensity versus molecular mass for both HC and LC. The percentage calculation was based on deconvoluted spectra of each chain. For the determination of % glycation in the LC, area of glycated LC was divided by sum of both areas from native and glycated LC. For the determination of % glycation in HC, area of glycated HC was divided by sum of both areas from native and glycated HC.

### Spiking study with highly fragmented CT-P6 sample

Various levels (1.63 – 7.54%) of fragmented CT-P6 samples were generated by spiking H2L1 enriched product. The level of total fragmentation in each sample was confirmed by non-reduced CE-SDS. The correlation between fragmentation level versus the relative anti-proliferation activity, FcγRIIIa-V and FcRn binding affinities were subsequently investigated.

### Spiking study with non-glycosylated CT-P6 sample

Various levels (0.58 – 37.53%) of non-glycosylated CT-P6 samples were generated by spiking fully aglycosylated product, prepared by treatment with PNGase F treatment, into control sample (0.58% non-glycosylation). The level of non-glycosylated product in each sample was confirmed by reduced CE-SDS. Correlation between non-glycosylation level versus the relative anti-proliferation activity, FcγRIIIa-V and FcRn binding affinities were subsequently investigated.

### HER2 binding affinity

HER2 ELISA was performed for EU-Herceptin®, CT-P6 drug product and US-Herceptin® to measure the binding affinity to HER2. Recombinant human ErbB2/Fc chimera (R&D systems, Cat. No. 1129-ER) was coated onto the 96-well plate and overnight incubated at 2-8°C. Samples were serially diluted from 500 ng/mL to 0.229 ng/mL (3-fold dilution, 8 points) and treated to the coated plates. The bound antibody was measured using HRP-conjugated goat anti-human gamma chain detection antibody (Sigma, Cat. No. A7164) followed by TMB treatment. After the stop reaction with sulfuric acid, the optical density values were measured at 450 nm / 650 nm. The relative HER2 binding affinity of sample was evaluated as (EC_50_ value of CT-P6 in-house reference standard / EC_50_ value of the samples × 100) using a 4-PL curve model by GraphPad Prism® software.

### Cell-based HER2 binding affinity

HER2 CELISA was performed for EU-Herceptin®, CT-P6 drug product and US-Herceptin® to measure the binding affinity of trastuzumab against HER2-overexpressing human breast cancer cell line, SK-BR3 cells were propagated onto the 96-well plate. After 2 days incubation, cells were fixed with 3.7% formaldehyde and then blocked with diluent buffer. Six-fold serial diluted sample (from 50,000 ng/mL to 0.18 ng/mL) was treated to the fixed cells. After binding, the cells were washed and subsequently incubated with HRP-conjugated anti-human kappa LC detection antibody (Sigma, Cat. No. A7164). TMB was treated to each well after the stop reaction with sulfuric acid, then the plate was measured at 450 nm / 650 nm. The relative cell-based HER2 binding affinity was determined using 4-PL logistic curve model by PLA 3.0 software.

### *In vitro* bioactivity (anti-proliferation activity using BT-474 cell)

The *in vitro* bioactivity assay was performed for EU-Herceptin®, CT-P6 drug product and US-Herceptin® to measure the anti-proliferation activity against the human breast cancer cell line BT474, which overexpress HER2 on the cell surface. These cells were incubated with EU-Herceptin®, CT-P6 drug product and US-Herceptin® at concentrations from 4,000 ng/mL to 7.813 ng/mL (serially 2-fold dilution, 10 points). After 5 days incubation, the bioactivity was determined by measuring BT-474 cell metabolic activity as an indicator of cell viability using CCK-8 assay (O.D 450 nm / 650 nm). The relative potency was evaluated using 4-PL curve model by PLA 3.0 software.

### C1q binding affinity

An anti-C1q ELISA was used to evaluate the binding affinity of EU-Herceptin®, CT-P6 drug product and US-Herceptin® to C1q complement. Samples were serially diluted from 60,000 ng/mL to 27.43 ng/mL (3-fold dilution, 8 points) and coated on a microplate surface. After blocking with CaCl_2_ assay buffer, 10 μg/mL of C1q (AbD Serotec, Cat. No. 2221-5504) was treated for 2 hours, and the bound C1q was measured using anti-C1q-HRP conjugate (AbD Serotec, Cat. No. 2221-5004P) followed by TMB treatment. After the stop reaction with sulfuric acid, the optical density values were measured at 450 nm / 650 nm. The relative C1q binding affinity of samples was evaluated as (EC_50_ value of CT-P6 in-house reference standard / EC_50_ value of the samples × 100) using a 4-PL curve model by GraphPad Prism® software.

### Fcγ receptor binding affinity

The Fcγ receptor binding affinity assays were performed using SPR to evaluate the binding affinity of EU-Herceptin®, CT-P6 drug product and US-Herceptin® to Fc receptors. Prior to the assay, each Fc receptor was diluted in 10 mM sodium acetate buffer, pH 5.0 and immobilized on the BIAcore CM5 chip using an amine coupling reaction. Any unstable immobilized Fc receptor was removed by at least 3 cycles (6 cycles for FcγRIIIa-F) of pre-run solution. Samples were serially diluted in HBS-EP buffer, pH 7.4, (HBS-EP, pH 6.0 for FcRn) and binding of the sample was measured in real time by the change in refractive index. The chip was regenerated in each cycle using regeneration solution appropriate for the Fc receptor. The relative binding affinity was evaluated as (K_D_ value of CT-P6 in-house reference standard / K_D_ value of the samples × 100) using steady state model by BIAevaluation software.

### Antibody-dependent cell-mediated cytotoxicity

ADCC activity of EU-Herceptin®, CT-P6 drug product and US-Herceptin®, which is mediated by effector cells through FcγR binding, was assessed using the calcein-AM release assay. The human breast cancer cell line SK-BR3, which over-expresses HER2 on the cell surface, was the target cell, and human PBMCs were used as the effector cells. Target cell was incubated with EU-Herceptin®, CT-P6 drug product and US-Herceptin® and PBMCs at E:T ratio (Effector cell:Target cell) of 16:1. Then, the cell cytotoxicity was measured by fluorescence values released calcein-AM and reported as ADCC activity and the relative ADCC activity of EU-Herceptin®, CT-P6 drug product and US-Herceptin® was determined as mean relative ADCC activity of each cytotoxicity at 3 concentrations (0.5, 1.3 and 3.2 ng/mL) within the linear range of ADCC response compared with that of CT-P6 in-house reference standard.

## Abbreviation

ADCC: antibody-dependent cell-mediated cytotoxicity
2-AB: 2-aminobenzamide
CD: circular dichroism
CDC: complement-dependent cytotoxicity
CELISA: cell-based enzyme linked immunosorbent assay
CE-SDS: capillary sodium dodecyl sulfate gel electrophoresis
CHO: Chinese hamster ovary
CI: confidence interval
DSC: differential scanning calorimetry
DTT: dithiothreitol
ELISA: enzyme linked immunosorbent assay
EM: equivalence margin
FT-IR: Fourier transform infra-red
HER2: human epidermal growth factor receptor 2
HILIC: hydrophilic interaction liquid chromatography
HPLC: high performance liquid chromatography
HMW: high molecular weight
HRP: horseradish peroxidase
IAM: iodoacetamide
IEC: ion exchange chromatography
IEF: isoelectric focusing
LC-MS-ESI: liquid chromatography mass spectrometry electrospray ionization
LMW: low molecular weight
MALS: multi-angle light scattering
NANA: N-acetylneuraminic acid
QR: quality range
RP: reference product
SEC: size-exclusion chromatography
SPR: surface plasmon resonance
SV-AUV: sedimentation velocity analytical ultracentrifugation
TMB: 3, 3′, 5, 5′-tetramethylbenzidine
UPLC: ultra-performance liquid chromatography
